# An Eccentricity Based Data Routing Protocol with Uniform Node Distribution in 3D WSN

**DOI:** 10.3390/s17092137

**Published:** 2017-09-16

**Authors:** A. S. M. Sanwar Hosen, Gi Hwan Cho, In-Ho Ra

**Affiliations:** 1 School of Computer, Information and Communication Engineering, Kunsan National University, Chonbuk 54150, Korea; sanwar@jbnu.ac.kr; 2 Division of Computer Science and Engineering, Chonbuk National University, Jeonbuk 54896, Korea; ghcho@jbnu.ac.kr

**Keywords:** wireless sensor network, three-dimensional space, data routing, network lifetime

## Abstract

Due to nonuniform node distribution, the energy consumption of nodes are imbalanced in clustering-based wireless sensor networks (WSNs). It might have more impact when nodes are deployed in a three-dimensional (3D) environment. In this regard, we propose the eccentricity based data routing (EDR) protocol in a 3D WSN with uniform node distribution. It includes network partitions called 3D subspaces/clusters of equal member nodes, an energy-efficient routing centroid (RC) nodes election and data routing algorithm. The RC nodes election conducts in a quasi-static nature until a certain period unlike the periodic cluster heads election of typical clustering-based routing. It not only reduces the energy consumption of nodes during the election phase, but also in intra-communication. At the same time, the routing algorithm selects a forwarding node in such a way that balances the energy consumption among RC nodes and reduces the number of hops towards the sink. The simulation results validate and ensure the performance supremacy of the EDR protocol compared to existing protocols in terms of various metrics such as steady state and network lifetime in particular. Meanwhile, the results show the EDR is more robust in uniform node distribution compared to nonuniform.

## 1. Introduction

Emerging in technology and sensor devices, the WSN range of usage in applications has been increasing globally such examples as Internet of Things (IoT) [1]. WSN consists of low-powered sensor nodes with sensing, computation and wireless communication capabilities. The constraints of sensor nodes pose a number of conceptual and optimization problems in different applications [2]. Numerous data routing and power management protocols have been proposed, where energy consumption is a vital issue.

Most of the researches on WSNs are still based on two-dimensional (2D) planes [3,4] while sensor node deployment in the real world may require 3D plane network structures. Such examples are ocean column monitoring [5], water quality monitoring [6], weather forecasting [7], climate monitoring [8] and so on, where the sensor nodes are required to be placed at different depths of the ocean and at different levels in the atmosphere, thus creating 3D WSNs. From these aspects, 3D WSN can be implemented in various potential applications in different environments. Primarily, minimizing the number of deployed nodes to achieve full-coverage and connectivity is important, because senor node deployment in 3D space is comparatively expensive. In this regard, several literatures have been studied addressing the optimization problem [9,10].

The major constraints of sensor nodes are the limited power and limited transmission range that require an energy-efficient routing strategy for a faraway BS or Sink. To resolve this issue, several data routing protocols have been proposed. Routing protocols based on clustering potentially are the most effective to reduce energy consumption of nodes [11] and have been widely accepted. The key strategy of this approach implies data gathering at a cluster head (CH) node from cluster members (CMs) and a hierarchical data forwarding towards the sink using single-hop or multi-hop depending on the network circumstances. Although this approach is delay tolerant, it is effective to apply a time division multiple access (TDMA) schedule to assign the time slots for the constituted nodes to communicate with each other and that can avoid the collisions of channels and interferences in the network. In addition, it guarantees reliable data communication with less packet loss, unlike other routing policies.

However, the performance of a clustering-based routing protocol in terms of network lifetime may vary upon the deployed nodes in different dimensions (such as in 2D or in 3D network fields). Besides, node distribution strategy (e.g., random and uniform/nonuniform or deterministic) depending on the applications is one of the key factors affecting the network lifetime. The random and nonuniform node distribution poses a network to form unequal size clusters in distributed clustering networks. Therefore, the energy consumption of nodes in different clusters is imbalanced and results in a shorter steady state and network lifetime.

In this paper, we propose the eccentricity based data routing (EDR) protocol [12] with uniform node distribution in a 3D WSN where the nodes are deployed in such a way that the prior divided 3D subspaces of the network contain equal numbers of member nodes. An eccentricity region based routing centroid (RC) node election in each individual subspace is applied that not only minimizes the intra-subspace communication costs but also balances the energy consumption of nodes. To minimize the cost of RC nodes election, a quasi-static period based RC nodes election is combined with periodic election. RC node during its quasi-static period continues as the RC that is defined by a threshold energy level. This policy continues until a certain period of time depending on the local circumstance of a subspace. It reduces the broadcasting of control messages which are economically viable considering the energy consumption of nodes. Once the residual energy of all member nodes in a subspace gets below the threshold level, then the RC node election is based on node’s fitness value that considers the factors of average distance of member nodes and residual energy of a node. This protocol utilizes the periodic data gathering at an RC node from its member nodes and hierarchical data forwarding from an RC node to the sink. An RC node selects an intermediate RC node that has a higher fitness value and minimizes the number of hops towards the sink. With the benefit of uniform node distribution, RC node concept and the proposed data forwarding strategy, the EDR protocol prolongs the steady state and the network lifetime adequately.

The rest of the paper is organized as follows. Section 2 reviews the related works. Section 3 presents the EDR protocol with the proposed network model and assumptions. Section 4 presents the performance evaluation. Finally, Section 5 concludes this work.

## 2. Related Works

A significant number of researches on routing protocol for WSNs have been proposed in the last few years. The low energy adaptive clustering hierarchy (LEACH) [13] is a pioneer work of clustering-based routing protocols in homogeneous WSNs and gives birth to many protocols. The idea of LEACH is clustering of nodes and periodic data gathering. For this, each node elects itself as a CH with a certain probability *p* and the role of CH rotates periodically among the nodes. To be a CH, each node generates a random number interval of 0 and 1. If the number is less than or equal to the threshold value *T*(*n*), it is elected as a CH. After the CH nodes election, each CH node broadcasts an advertisement message over the network within a radio range *R*. Upon receiving the advertisements from the elected CHs, a nearest CH node is selected to form a cluster by calculating the distance followed by sending a join message to the selected CH. A CH node then fixes the TDMA schedule and broadcasts the message to its CMs. A CH node acts as a local BS that receives and aggregates data from CMs and sends the aggregated data directly (single-hop) to the BS. Once the set of nodes *C* is elected as CHs at time *t*_i_, in next round, a new set *C'* is elected as CHs at time *t*_i+d_. Although it distributes the energy consumption of nodes equally, it leads to additional routing overhead, resulting in excessive use of limited energy of a CH node due to unequal CMs. Its single-hop communication strategy constructs the network less scalable. Meanwhile, it does not consider the residual energy of nodes in CHs election that cause a shorter steady state and network lifetime.

Inspired from LEACH, many protocols have been proposed to improve the network lifetime. EADEEG [14] is a novel distributed clustering-based routing algorithm. It considers the ratio of the residual energy of neighbor nodes and the residual energy of the node itself during a CH node election. It achieves equal CHs distribution over the network, which prolongs the network lifetime. The drawbacks of this protocol are less scalable as LEACH and it cannot deal with the ‘*isolated points*’ during inter-cluster communication in some cases. To solve this problem, a distributed energy saving clustering and routing algorithm called BPEC has been proposed in [15]. It elects a CH node by two different quality functions such as primary probability and subsidiary probability. Its primary probability function is similar to EADEEG and the subsidiary probability considers the node’s degree in addition. It can solve the ‘*isolated points*’ unlike the EADEEG and can keep all the CH nodes connected during inter-cluster communication. To extend the network lifetime a degree further, a lifetime maximization algorithm has been proposed in [16]. It considers residual energy of nodes and the required transmission energy of path towards the BS during CH nodes election. It distributes the load of the nodes almost evenly, so that the energy consumption of the nodes is distributed equally. In [17], the authors proposed a *k*-connected overlapping method in clustering. It selects CH nodes on the basis of available energy status of the nodes that enhances the network lifetime. [18] is a link aware clustering-based routing algorithm. It adopts a clustering metric based on predicted transmission count, unlike considering residual energy in CH nodes election. This method determines a reliable and energy-efficient path in routing to prolong the network lifetime.

In [19], the authors proposed an energy-aware distributed clustering (EADC) based routing protocol that can prolong network lifetime significantly. To distribute the CHs equally over the network, it partitions the network into CHs competition regions defined by *R*_c_. CH nodes are elected in each of the regions, so that the clusters have the approximate number of members and coverage area. It considers the residual energy of nodes during CH nodes election like as EADEEG and forms clusters, which is similar to LEACH. Besides, the authors extended the protocol for single-hop to multi-hop communication. To select a route towards the BS, each CH node broadcasts a route message within the radio range *R*_r_ = 2*R*_c_. It selects a CH node as an intermediate node based on some factors, such as the minimum number of member nodes obtained cluster, a higher residual energy obtained CH and a minimum distance of CH node from the BS. The CH node which satisfies the factors is selected as a data forwarding node. However, its CH nodes election policy encourages broadcasting an additional number of control messages in each round. It incurs a large control message overhead that consumes additional energy of nodes and results in significant effects on the network lifetime. Meanwhile, its data forwarding strategy influences a data message to traverse from a CH node to the BS through the more hops in the network and consumes additional energy of the constituted nodes.

The proposed EDR differs from the above mentioned protocols due to its unique RC nodes election and data routing policy. It emphasizes to minimize the overall data routing cost which includes minimum intra-subspace and inter-subspace communications that prolongs the steady state and network lifetime. Although the LEACH and EADC protocols are proposed in 2D WSNs with nonuniform node distribution, we are interested in implementing those protocols in a 3D WSN named as 3D LEACH and 3D EADC with uniform node distribution. Moreover, we are obliged to exhibit the performance of our proposed protocol in comparison with 3D LEACH and 3D EADC in terms of steady state and network lifetime.

## 3. Proposed EDR Protocol

### 3.1. Network Model

The proposed protocol considers the following network model of an area coverage. A sink can be located anywhere within the communication range from the network field. Sensor nodes *n* are dispersed randomly and uniformly in the unit cube of volume *V* according to the *X*, *Y* and *Z* coordinates. To simplify our generalized idea, a 3D WSN data routing model has been designed based on a few rational assumptions as follows. The space is divided into 3D subspaces which are ordered sequentially in different levels *l*_i_, *i* = {1,2,3,…,*h*} according to the location of sink in the network field. All the nodes and the sink are stationary after deployment. The nodes are homogeneous and each node has a unique identity (*id*). Unlike the nodes, the sink has no energy constraint. The nodes know about their positions’ coordinates (*x*,*y*,*z*) as well as being familiar with the coordinates of the edges of the subspaces. Several sensor node localization methods have been proposed based on global positioning system (GPS), received signal strength (RSS) in 3D WSNs [20,21]. Here, a Cartesian coordinate system is used to represent the position of a node that can be obtained from a GPS module. The GPS module calculates the position of each node and it will be used at the time of node deployment. After that, it will be switched off to save energy. During network operation phase, different types of messages are used in Table 1.

### 3.2. Number of Subspaces

According to the EDR, the network field is divided into a number of predefined subspaces in different levels *l.* The number of subspaces *k* formation is defined in Equation (1):

(1) $$SubSL=\frac{SD}{l\surd 3},\;k=\;\frac{V}{SubSL^{3}},$$

where *SubSL* is the edge length of a unit cube 3D subspace, *l* denotes a number of levels and *SD* is the space diagonal length of the network field.

As the nodes are uniformly distributed, each subspace contains an equal number of nodes (*n*/*k*). A cluster is formed among the constituted nodes in a subspace and the size of the cluster is fixed.

### 3.3. Information Collection Phase

This phase starts at predefined time *T*_1_. During this time, each node broadcasts its status along with a *Hello_Msg* within its radio range *R*_s_, where *R*_s_ is equal to the subspace diagonal length. The nodes belonging in the same subspace receive the message and list the information in their members’ distance table (MDT) that contains [*node_id*, *subspace_id*, *node_distance*]. From the distance information of the member nodes, each node (e.g., a node *s*_i_ in a subspace) computes its members’ average distance (*MAD*) that is defined in the following Equation (2):

(2) $$MAD=\frac{1}{m-1}\overset{m-1}{\underset{j=1,j\neq i}{\sum }}d(s_{i},s_{j}),$$

where *m* is the number of member nodes in a subspace and *d* is the distance of a member node *s*_j_ of *s*_i_.

After completing the *MAD* calculation, each node broadcasts it along with a *Node_Msg* within its radio range *R*_min_, where *R*_min_ is equal to the maximum distant member node in its MDT. Nodes in the same subspace receive the message and list the information in their members’ average distance table (MADT) that contains the attributes [*node_id*, *subspace_id*, *average_distance*, *residual_energy*, *node_type*, *node_fitness*, *flag*]. Initially, all nodes are typed normal ‘*N*’ and the *node_fitness* and *flags* values are 0. The details of this phase is given by the pseudo-code in Algorithm 1.
**Algorithm 1: Information collection algorithm****start** *state ←* Member node **while** (*T*_1_ has not expired) **do**  Broadcast *Hello_Msg*  Receive *Hello_Msg*  List in a MDT  Broadcast *MAD* with *Node_Msg*  Receive *Node_Msg*  List in a MADT **end****end**

### 3.4. RC Nodes Election Phase

After completing the network initialization phase, the RC nodes election starts at *T*_2_ time. In this phase, each node finds the minimum average distance obtaining node from its own MADT. If the minimum *MAD* obtaining *node_id* match with its own *id*, it broadcasts a *Schedule_Msg* according to the member nodes from its MADT within its radio range *R*_min_. Otherwise, it will wait to receive the *Schedule_Msg* from another minimum *MAD* obtaining member node in the same subspace. Meanwhile, the self-elected RC node updates its own MADT by changing its initial *node_type* from ‘*N*’ to ‘*RC*’ and *flag* bit from 0 to 1. Upon receiving the *Schedule_Msg*, each member node updates its own MADT by changing the initial *node _type* and *flag* bit of the elected RC node according to the index of that node. Once a node is elected as an RC, it will continue its role as the RC until its residual energy gets as below as the threshold energy level *T*_E_, which is defined by *Ei*/*α*, where *Ei* is the initial energy of a node and *α* > 0 is a factor value.

When an RC node’s residual energy is less than or equal to the *T*_E_, after completing the data forwarding task, it broadcasts a *Not_Msg* within its radio range *R*_min_ and updates its own *node_type* and *flag* bit back to ‘*N*’ and 0, respectively. On receiving the message, each member node updates its own MADT by changing the *node_type* and *flag* bit according to the index of the RC node and repeats the RC node election within the particular subspace. In this case, whenever all the nodes in a subspace have already participated as the RC node, the residual energy of each of the nodes will be less than the *T*_E_. In this scenario, the RC node election in the subspace will be based on the node’s fitness value *F*_1_ defined in Equation (3) in a periodic fashion. The equation guarantees that the node which has less average distance and higher residual energy has a higher fitness value. For this, the nodes broadcast their estimated *F*_1_ along with their *Node_Msg*s. The member nodes receive the messages and update their MADT. A higher *F*_1_ obtained node will be elected as an RC node in each round.

(3) $$F_{1}=\;\beta \frac{s_{i}.Er\in SubS_{k}}{\sum_{j=1}^{m\in SubS_{k}}s_{j}.Ei}+\left(1-\beta \right)\frac{1}{s_{i}.MAD},$$

where *s*_i_.*Er* is the residual energy of a node in a subspace *SubS*_k_, *s*_j_.*Ei* is the initial energy of a node in the same subspace, *m* is the number of member nodes in that subspace, and *β* is the weight factor value between 0 and 1.

The details of this phase is given by the pseudo-code in Algorithm 2.
**Algorithm 2: Routing centroid (RC) nodes election algorithm****start** *state ←* Candidate and RC node election **while** (*T*_2_ has not expired) **do**  **if**
*s*_i_.*type* == ‘*RC*’ & *s*_i_.*Er* > *T*_E_
**do**   Continue as an RC node    **if**
*RC*_i_.*Er* ≤ *T*_E_
**do**    Broadcast *Not_Msg*    Update own MADT    Receive *Node_Msg *(member nodes)     Update own MADT (member nodes)   **end**  **else if**
*s*_i_.MADT(:,7) == Null & *s*_i_.*Er* > *T*_E_
**do**    Minimum *MAD* obtaining node is selected as an RC node from MADT     Update own MADT    Broadcast *Schedule_Msg*    Receive *Schedule_Msg* (member nodes)    Update MADT (member nodes)  **else if**
*s*_i_.MADT(:,4) < *T*_E_
**do**    Broadcast *F*_1_ value with *Node_Msg*    Receive the *Node_Msg* (member nodes)    Update own MADT    Maximum *F*_1_ obtaining node is selected as an RC node from MADT    Broadcast *Schedule_Msg*    Receive *Schedule_Msg* (member nodes)  **end** **end****end**

### 3.5. Data Communication Phase

**Intra-Subspace Communication.** Once the RC nodes election phase is completed and the TDMA schedule is fixed, the local data transmission can begin at time *T*_3_. During the allocated time, a member node sends its local information (*D_Msg*) to the corresponding RC node within the radio range *R*_min_ (the distance between a member node and the RC node in a subspace). The radio of each non-RC-node is turned off until the node’s allocated transmission time. The receiver of the RC node should be on to receive all the data from the member nodes in a subspace.

During this communication, if a member node has not transmitted data to its RC node within its allocated transmission time due to the death of that node or for any other reasons, it will be listed as a missing node. The missing node(s) is considered as a blacklisted node. An RC node checks the missing node(s) and updates its own MADT by deleting the blacklisted node(s). Meanwhile, it broadcasts about the missing node(s) to its member nodes along with the *Not_Msg*. On receiving the message, the member nodes update their MADT by deleting the blacklisted node and it will be excluded out of the network from the rest of the process. After the data has been received, an RC node performs the signal processing function to compress/fuse the data into a single packet called aggregated data (*AD_Msg*). Once the data fusion process is completed, an RC node transmits the *AD_Msg* to a next hop which can be the sink or a forwarding RC node with a higher fitness value.

**Inter-Subspace Communication.** This phase starts at predefined time *T*_4_ in each round. In this phase, each RC node broadcasts its status along with a *Route_Msg* within its radio range *R*_max_. The *R*_max_ = 2*R*_s_ is defined as the maximum radio transmission range of a node in the network. For the fitness value, each RC node computes its *F*_2_ which is defined in Equation (4). The equation guarantees that the RC node of large member nodes obtains a higher residual energy and has a higher fitness value.

(4) $$F_{2}=\;\beta \frac{RC_{k}.Er}{\sum_{i=1}^{m\in SubS_{k}}s_{i}.Ei}+\left(1-\beta \right)\frac{RC_{k}.m}{n},$$

where, *RC*_k_.*Er* is the residual energy of an RC node in a subspace *k*, *s*_i_.*Ei* is the initial energy of a member node in the subspace, *RC*_k_.*m* is the number of member nodes of that subspace and *n* is the total deployed nodes in the network.

An RC node receives the *Route_Msg*s from other RC nodes in two different cases.

**Case 1:** An *RC*_i_ node in level *l*_i_|(1 ≤ *l*_i_ < *l*_h_) receives the message from an *RC*_k_ node in different levels *l*_k_|(*l*_i_ < *l_k_*≤ *l*_h_) within the range *R*_max_ and belongs to the volume of the region $v_{RC}^{\prime }$ which is defined in Equation (5):

(5) $$v_{RC}^{\prime }={\left(X=Y\right)}^{2}\left(l_{hz}^{u}-l_{iz}^{u}\in SubS_{k}\left(RC_{k}\right)\right),$$

where *l*^u^ is the upper value of the *z*-coordinate (height) of a level *l*_(.)_, *SubS*_k_ is the subspace of an *RC*_k_ in a level *l*_k_, *k* > *i*. The factors *X* and *Z* are defined based on the location of the sink.

**Case 2.** An *RC*_i_ node receives the message from an *RC*_k_ node in the same level where *l_i_* = *l_h_* and belongs to the volume of the region $v^{\prime \prime }{}_{RC}$ which is defined in Equation (6):

(6) $$v_{RC}^{\prime \prime }=X^{2}l_{hz}.$$

**Figure 1:**
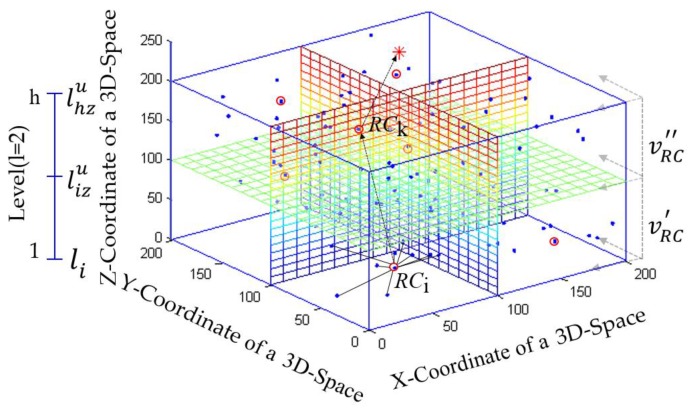
An example of forwarding routing centroid (RC) node selection.

On receiving the messages, the information of other RC nodes is listed in its neighboring RC nodes’ table (NRCT) which contains [*node_id*, *subspace_id*, *node_type*, *node_fitness*, *dist_sink*]. Generally, an RC node first checks the threshold distance (*T*_D_) between the sink to communicate directly to the sink. If the distance between the sink *d*(*RC*_i_,s*ink*) is greater than the *T*_D_, then the RC node selects a higher *F*_2_ obtaining RC node as a forwarding node from its NRCT. If there is more than one RC node that has the largest *F*_2_, the RC node selects the one with a minimum distance RC node from the sink. In this case, if there is no RC node in its NRCT and the sink is within the *R*_max_, then it transmits the *AD_Msg* directly to the sink. A forwarding RC node receives an *AD_Msg* from the other RC node and it forwards the message directly to its next hop or to the sink without fusion. An illustration of forwarding RC node selection and data forwarding towards the sink is shown in Figure 1. The details of this phase is given by the pseudo-code in Algorithm 3.
**Algorithm 3: Inter-subspace data communication algorithm****start** *state ←* Data communication   Broadcast *Route_Msg* (RC nodes)  **if**
*d*(*RC*_j_,*sink*) ≤ *T*_D _**do**   *nexthop ← sink*  **end**  **while** (*T*_4_ has not expired) **do**   **if**
*s*_i_.*type* == ‘*RC*’ **do**    Receive *Route_Msg*    Update own NRCT   **end**   **if**
*s*_i_.*type* == ‘*RC*’ & *d*(*RC*_i_,*sink*) > *T*_D_
**do**    **if**
*RC*_i_.NRCT(:,3) == ‘RC’ & size(*RC*_i_.NRCT(:,1)) != Null **do**     Select a higher *F*_2_ obtaining *RC*_k_     *nexthop ←RC*_k_    **else if**
*d*(*RC*_i_,*sink*) ≤ *R*_max_
**do**      *nexthop ←sink*    **end**   **end**  **end****end**

## 4. Performance Evaluation

### 4.1. Simulation Setup

The performance of EDR is evaluated with random and uniform node distribution in MATLAB 9.2.0.556344 (R2017a). It is assumed that all of the deployed nodes are constituted in such a way that there are always other nodes within their radio range *R*_s_. The parameters used in the simulation are shown in Table 2.

In the simulation, the transmitting and receiving costs are measured based on the first order radio dissipation model that has been described in [13]. In this model, the transmitter and receiver dissipate *E*_elce_ to run the transmitter and receiver circuitry. To amplify the signal, amplifier dissipates *E*_amp_ or *E*_fs_. Equations (7) and (8) were used to calculate the energy in transmitting and receiving an *L*-bits message over a distance *d* between the transmitter and the receiver. To aggregate data, an RC node consumes energy defined by *E*_DA_.

(7) $$E_{Tx}\left(L,d\right)=\{\begin{array}{cc} E_{elec}L+E_{fs}Ld^{2} & if\;d\leq d_{0}\\ E_{elec}L+E_{amp}Ld^{4} & if\;d>d_{0} \end{array},$$

where *d*_0_ is defined by $d_{0}=\;\sqrt{\frac{E_{fs}}{E_{amp}}}.$

(8) $$E_{Rx}=\;E_{elec}L.$$

The number of RC/CH nodes election in EDR and 3D EADC are fixed during each round that are prior defined based on the number of subspaces/competition regions of the CH nodes election, respectively. By contrast, the number of elected CH nodes in 3D LEACH differs from EDR and 3D EADC; it fluctuates randomly instead of the fixed numbers. The subspaces of EDR and the competition regions of 3D EADC contain an equal number of member nodes that is (*n* = 120)/(*k* = 8) = 15.

The EDR protocol is compared with 3D LEACH and 3D EADC in the same network field in terms of steady state and network lifetime. In the simulation, the parameters used for 3D LEACH and 3D EADC are: radio range *R* for the advertisement of the elected CH node to form a cluster that is equal to *R*_max_, CH competition range *R*_c_ is equal to *R*_s_, the radio range *r* for broadcasting residual energy to neighborhood nodes is 100 m and the range *R*_r_ for broadcasting route message is equal to 2*R*_s_, respectively. An average percentage of nodes to become the CHs in each round in 3D LEACH is *p* = 0.1.

**Figure 2:**
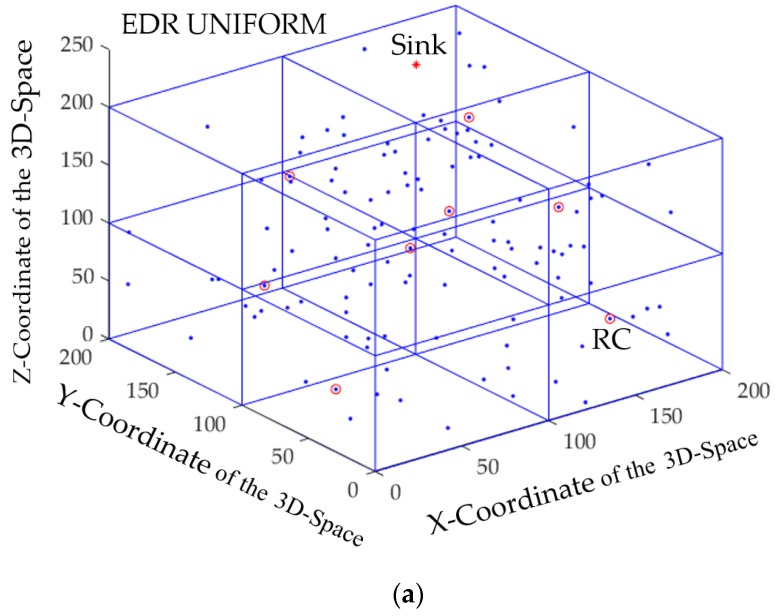

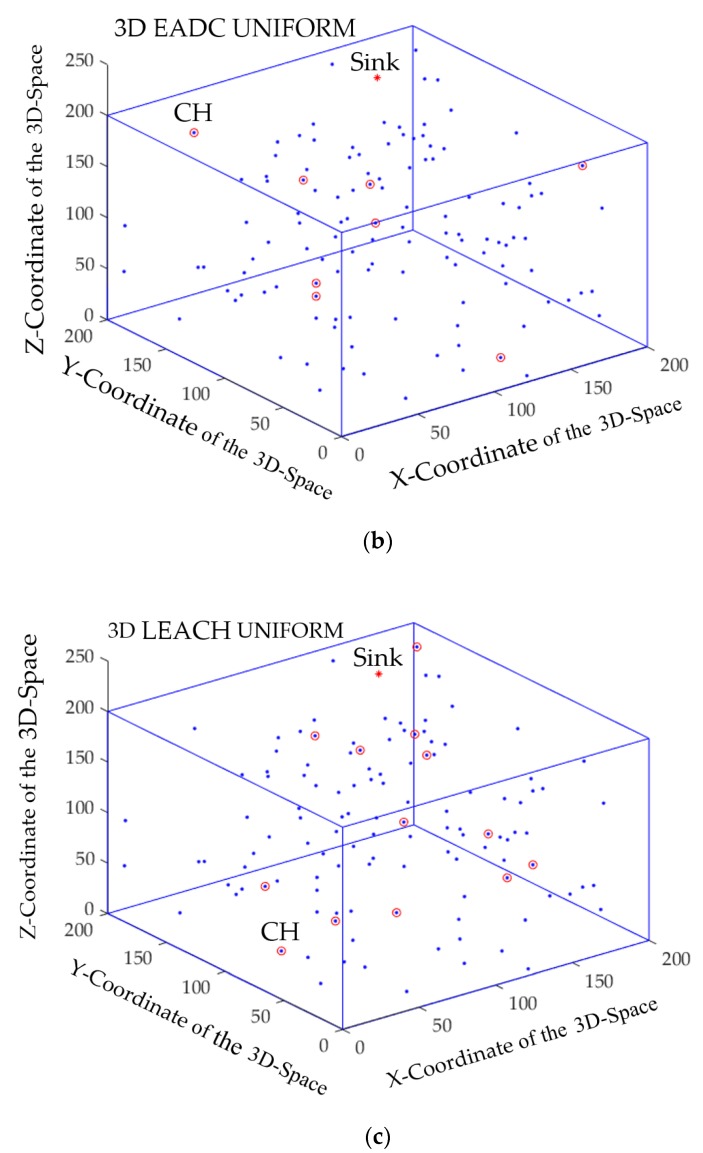
Elected RC/cluster head (CH) nodes in the three-dimensional wireless sensor network (3D WSN) field of: (**a**) eccentricity based data routing (EDR);(**b**) 3D energy-aware distributed clustering (3D EADC); (**c**) 3D low energy adaptive clustering hierarchy (3D LEACH).

During the simulation, the CH nodes and the RC nodes were elected at a certain round in the protocols are shown in Figure 2. The figure shows that the number of elected CHs in 3D LEACH is random and the number of RCs/CHs is predefined in EDR and 3D EADC, respectively.

### 4.2. Simulation Results

#### 4.2.1. Number of RCs/CHs Change

**Figure 3:**
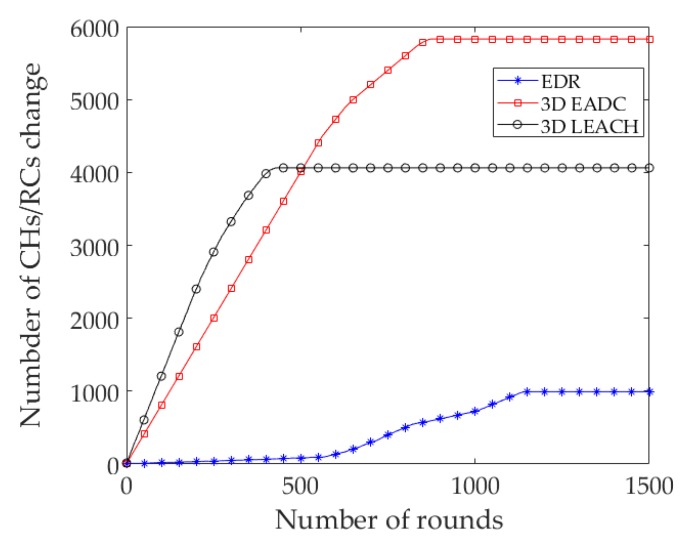
The CH/RC nodes changed at different rounds.

There are no changes of RC nodes until the first 38 rounds in EDR, as shown in Figure 3. The changing rates increase gradually in a quasi-static nature. Meanwhile, the figure shows a node participated as an RC/CH node an average of 7.78 times, 48.58 times and 33.85 times throughout the network lifetime of EDR, 3D EADC, and 3D LEACH, respectively.

The cluster formation approaches of LEACH and EADC are like forming the Voronoi cell [22] in a 2D WSN. In case of the 3D WSN, the cluster formation of 3D LEACH and 3D EADC requires the 3D Voronoi cell or convex hull approach. In this approach, a CH node may have a higher probability of taking control of a large number of member nodes as CMs. It results in an enormous data aggregation load of this CH node. The consequence is the premature death of the CH node due to the huge energy dissipation. In this context, the CH nodes election of 3D EADC is more advanced than that of 3D LEACH.

#### 4.2.2. Number of Control Packets

**Figure 4:**
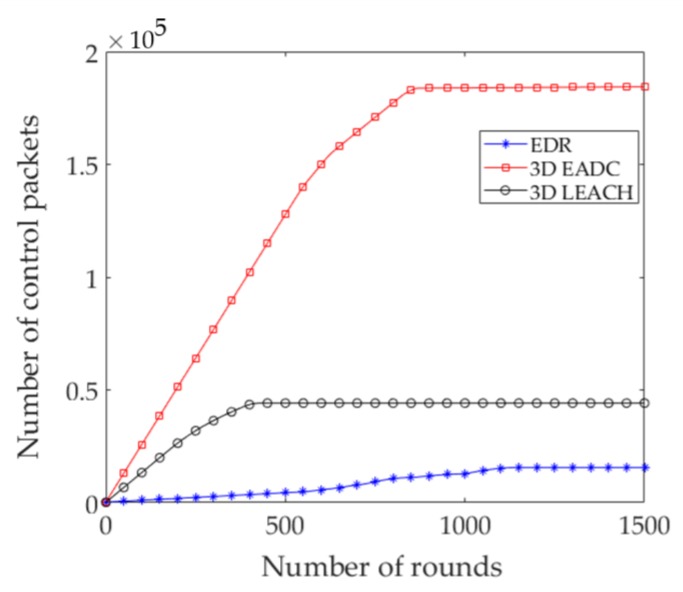
Number of control packets transmitted in the network.

Reducing the number of control packets during the network setup phases is a challenging task to design a cost effective routing model. The proposed quasi-static nature of RC nodes election reduces a significant number of control packets throughout the network lifetime. In order for nodes to be elected as the CHs, the 3D EADC requires the collecting of information about their residual energy at each round. This encourages transmitting and receiving an additional number of packets and specifically, the number will increase with the higher number of neighbor nodes within a range *R*_c_. Figure 4 shows the number of control packets transmitted in the network. EDR reduces the control packets 64.65% and 91.53% throughout the network lifetime of 3D LEACH and 3D EADC, respectively.

#### 4.2.3. Throughput

**Figure 5:**
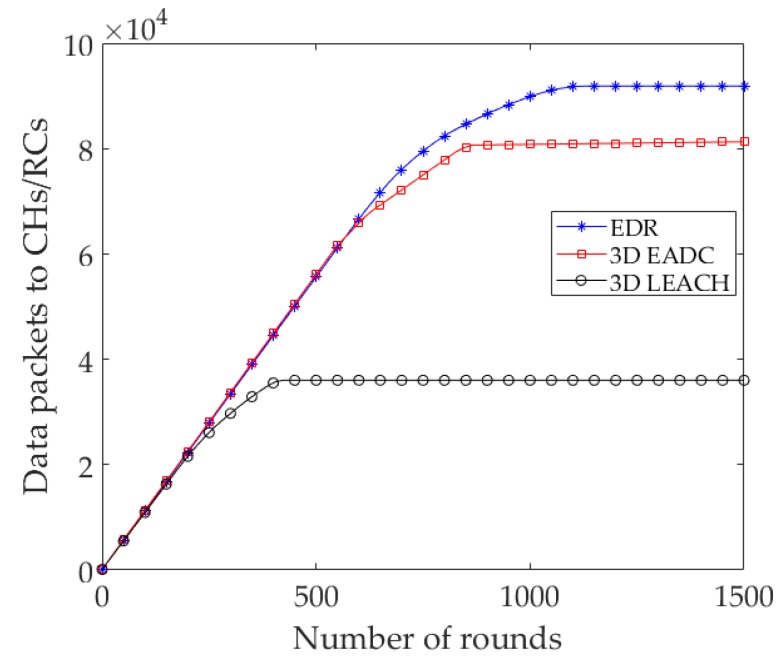
Number of local data packets sent to CH/RC nodes.

Throughput is defined as the total number of data packets successfully received at sink. In this context, the number of *Ad_Msg*s received at the sink are considered as the throughput. In clustering and data fusion based approaches, local data has a significant role in information gain of the network. Therefore, we have taken into consideration the received *D_Msg*s at different RC/CH nodes, partially. Figure 5 shows that the number of *D_Msg*s received by RC/CH nodes of the EDR and 3D EADC is higher compared to 3D LEACH. Meanwhile, the results show that the number of the packets decreases due to the premature death of nodes in the network of 3D LEACH. However, the EDR outperforms 3D LEACH and 3D EADC, considering the total number of *D_Msg*s received at RC nodes throughout their network lifetime.

**Figure 6:**
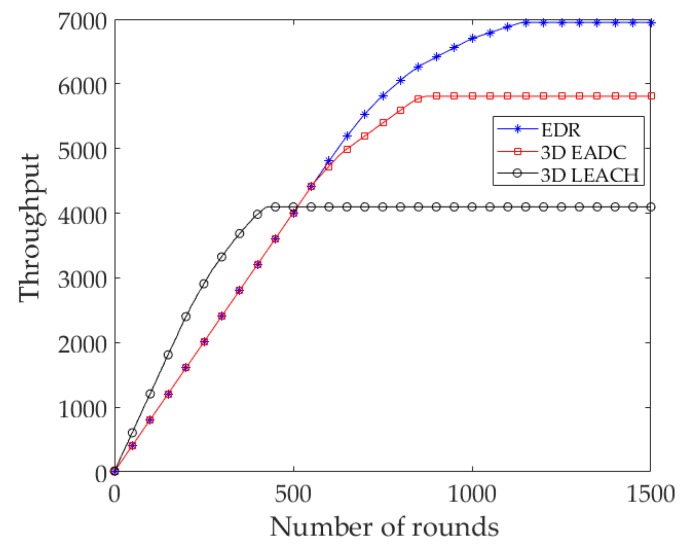
Number of data packets sent to the sink from CH/RC nodes.

Figure 6 shows the throughput of the protocols. The throughput of EDR is larger than that of 3D LEACH and 3D EADC, considering the network lifetime of those protocols. Throughout the network lifetime of 3D LEACH, the throughput of 3D LEACH is larger than the EDR and 3D EADC, because the 3D LEACH elects an average of *p*×*n*|( *p* = 0.1, *n* = 120) = 12 CHs in each round, unlike the predefined 8 RCs/CHs in each round in EDR and 3D EADC.

#### 4.2.4. Number of Hops

**Figure 7:**
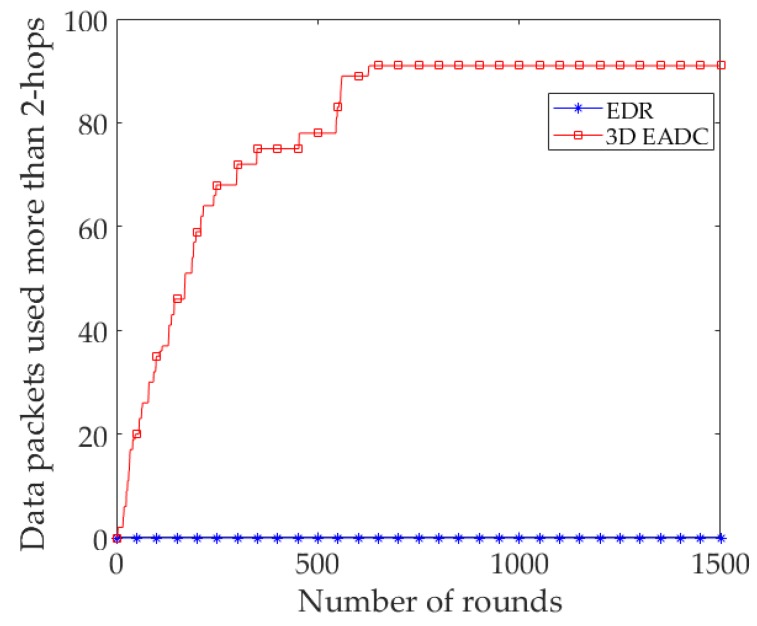
Data packets received at the sink used 3-hops.

Figure 7 shows the number of packets (*AD_Msg*) used 3-hops to traverse from an RC/CH node to the sink. EDR reduces the number of hops significantly during the data forwarding from an RC node to the sink. It reduces a significant number of the packets of 3D EADC in consideration of using 3-hops distance from an RC node to the sink.

#### 4.2.5. Network Lifetime

The nature of energy depletion of the constituted nodes in the protocols are distinctive. The energy consumption of the nodes in EDR is not equally distributed; rather, the elected RC nodes dissipate energy faster than the normal nodes until the defined *T*_E_ level. Contrasting with EDR, energy depletion of the nodes tends to be distributed in 3D LEACH and 3D EADC. The energy consumption of the constituted nodes in 3D EADC is more equally distributed compared to 3D LEACH.

**Figure 8:**
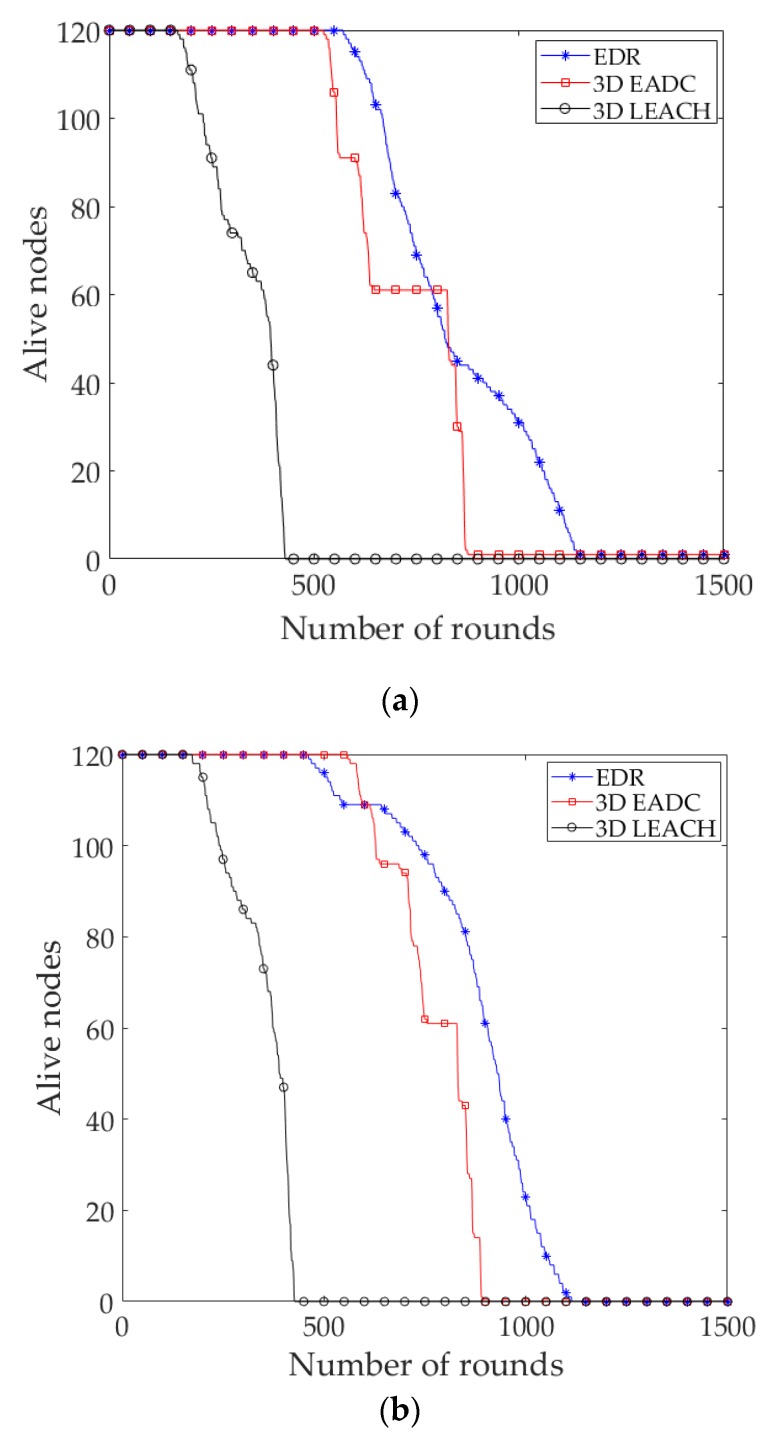
(**a**) Number of alive nodes in each round (uniform); (**b**) Number of alive nodes in each round (a best case of nonuniform node distribution where *α* = 4 and the nodes are not equally distributed over the network).

The lifetime of a WSN is defined by a certain percentage of live nodes in EADC. Here, the network lifetime is defined as the time until 10% of nodes are alive. Figure 8a compares the network lifetime in uniform node distribution, considering the alive nodes in the network. The result shows that the steady state and network lifetime of EDR are longer than the 3D LEACH and the 3D EADC. The steady state of a network is defined as the time period of the first node die (FND) in the network. EDR outperforms on averages of 1.34 times, 1.09 times and 2.59 times, 1.26 times of the steady state and network lifetime of 3D LEACH and 3D EADC, respectively.

To compare the network lifetime of uniform with nonuniform node distribution, we also simulated the protocols with nonuniform node distribution in the same network circumstance. Figure 8a,b clearly depict that the steady state and network lifetime of EDR are enhanced in uniform compared to nonuniform. It is worth noticing that there is no significant changes of the network lifetime of 3D LEACH and 3D EADC with different node distributions.

#### 4.2.6 Impact of *T*_E_ on Network Lifetime

The factor *α* to define *T*_E_ plays a significant role in network lifetime of EDR. It must be considered in such a way that whenever all the member nodes in a subspace are below *T*_E_, they can continue with the rest of periodic RC nodes election process in the subspaces. Otherwise, the overall network lifetime may decrease due to the lack of insufficient residual energy of the nodes after participating in the RC nodes election in their quasi-static periods.

**Figure 9:**
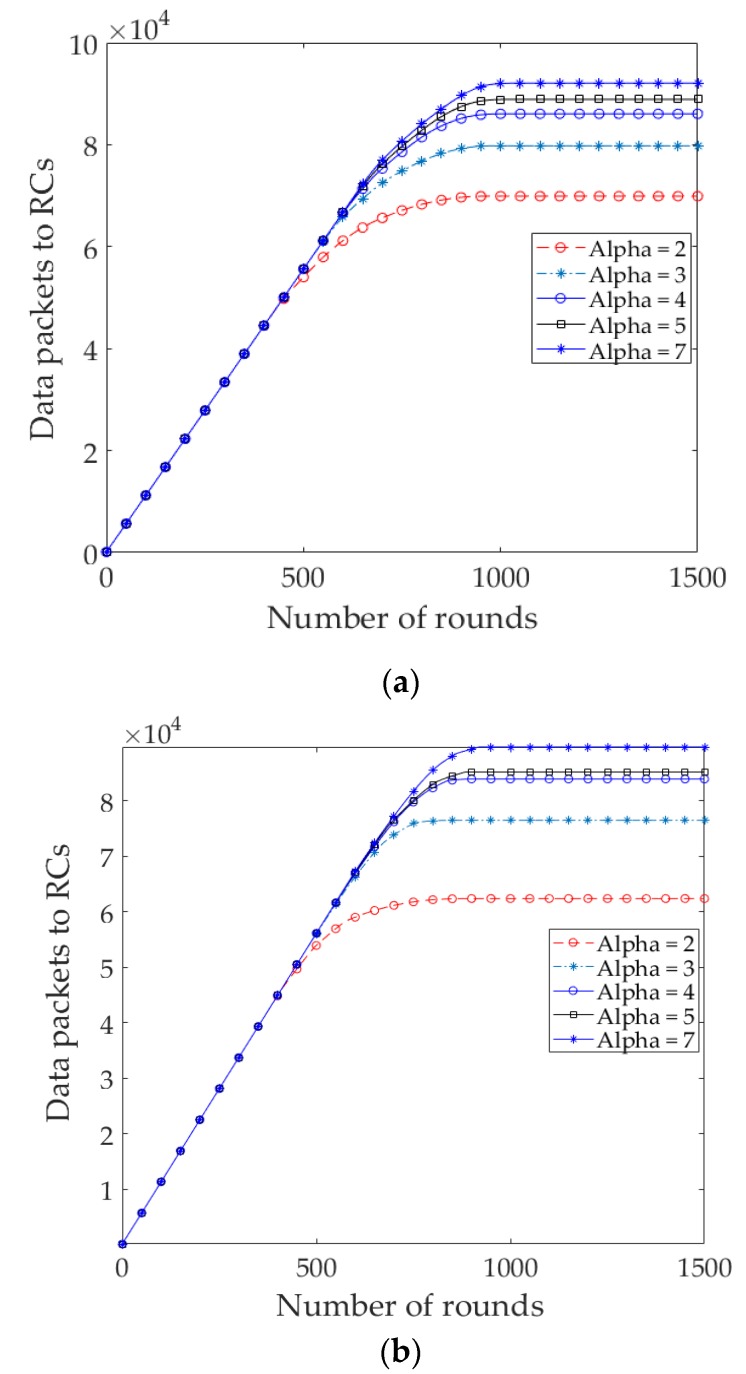
(**a**) Number of local data packets sent to RC nodes for different *T*_E_ in EDR (uniform); (**b**) Number of local data packets sent to RC nodes for different *T*_E_ in EDR (nonuniform).

**Figure 10:**
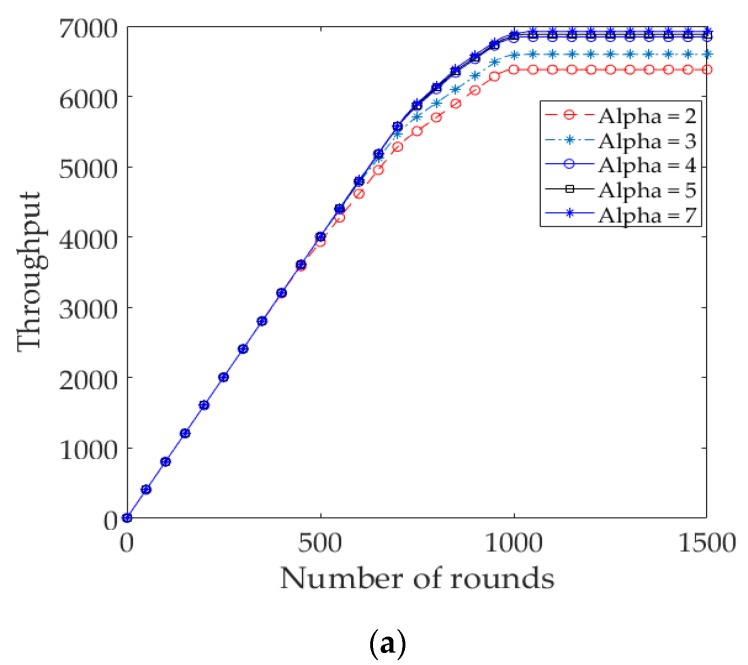

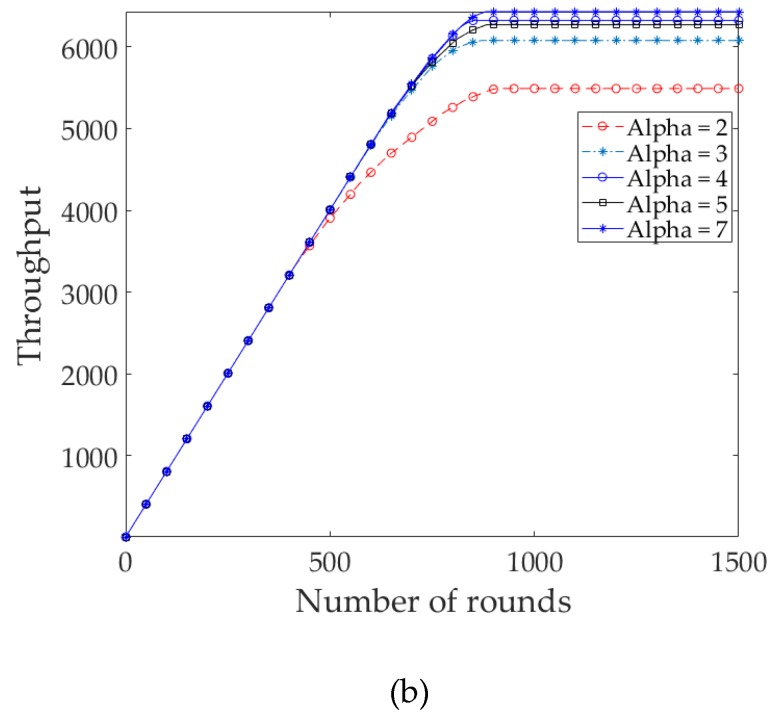
(**a**) Number of data packets sent to the sink for different *T*_E_ in EDR (uniform); (**b**) Number of data packets sent to the sink for different *T*_E_ in EDR (nonuniform).

**Figure 11:**
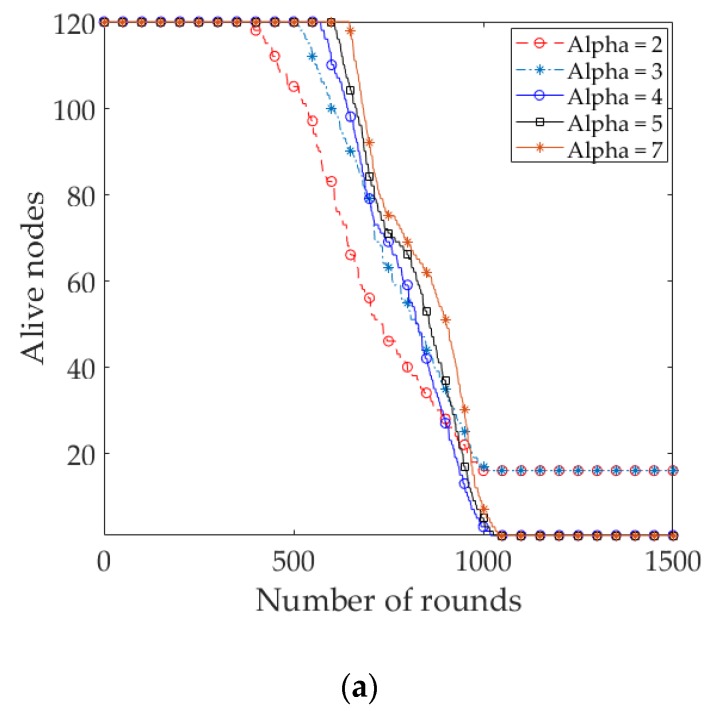

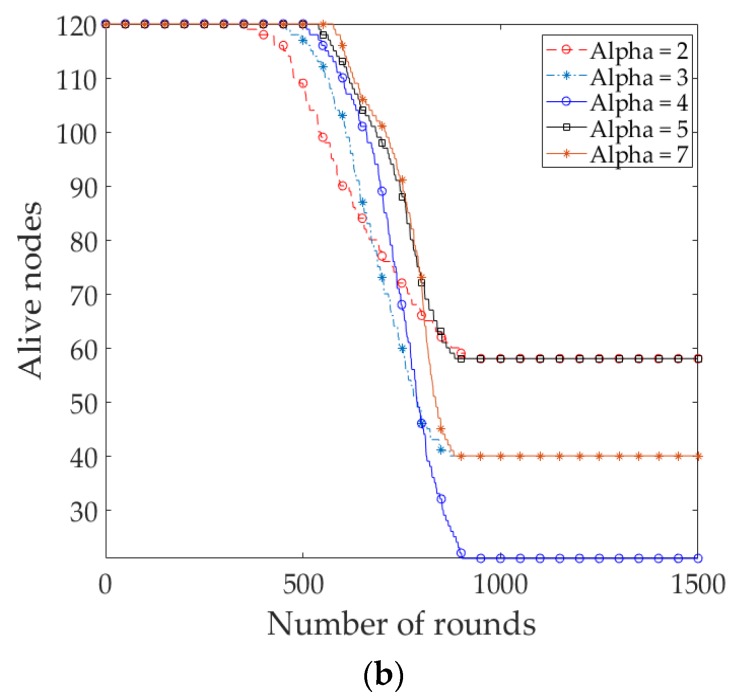
(**a**) Number of alive nodes at each round for different *T*_E_ in EDR (uniform); (**b**) Number of alive nodes at each round for different *T*_E_ in EDR (nonuniform).

We examine the impact of *T*_E_ on the network lifetime of EDR for different values of *α* in both of the node distributions. Besides the network lifetime, we took into consideration the number of *D_Msg*s and throughput received at RC nodes and sink, respectively. Figure 9a and Figure 10a show the number of packets received at different RC nodes and sink, accordingly in uniform node distribution. The results show that the number of local packets received at RC nodes and throughput increase with increases in the value of *α*. It is observed that, as the value of *α* increased (*α* > 7), the number of packets is decreased significantly due to the shorter steady state and network lifetime. Figure 9b and Figure 10b show the packets received in the nonuniform node distribution. It is to be noticed that when *α* is greater than a certain value (i.e., *α* = 4), the throughput increases, but some of the nodes are being isolated from the sink, resulting in a shorter network lifetime, as shown in Figure 11b:

The steady state as well as the network lifetime of EDR enhances with the increase in the value of *α* in uniform compared to nonuniform, as shown in Figure 11a,b, respectively. The distinctive feature of the uniform to notice that when the value of *α* is smaller (*α* = 2, *α* = 3), around 20% of nodes in the distant subspaces are being isolated from the sink due to the early death of the nodes in the nearest subspaces to sink. As the nodes are equally distributed over the network and the quasi-static period is shorter, the periodic RC nodes election period gets longer. Therefore, the nodes dissipate energy more quickly due to the process of RC nodes election in each round. Moreover, the elected RC nodes near the sink participate as the data forwarding nodes for the distant RC nodes in the network and consume more energy compared to the distant RC nodes which results in the nearest nodes to the sink to die more quickly. On the other hand, when the value of *α* gets larger, the quasi-static period is getting longer; thus, the periodic RC nodes election period is getting shorter. The consequence is that the nodes save energy due to a minimized number of RC nodes election processes throughout the network lifetime. As a result, the steady state and the network lifetime are prolonged.

### 4.3. Discussion

Through the simulations, some fundamental factors are measured that are essential to design an energy-efficient routing protocol. The simulation results reveal that the randomness in the CH nodes election and a number of CHs in each round of the clustering-based approach, as in 3D LEACH, effect the overall network energy consumption due to unawareness about the intra-cluster communication cost of the network.

A hierarchical multi-hop data forwarding strategy is more effective than the single-hop, considering from a CH node to a faraway sink. Choosing a forwarding node depends on some parameters without considering that the position of the node towards the sink may increase the number of intermediate nodes in the data forwarding, as in 3D EADC. This causes a higher inter-cluster communication cost. To resolve this problem, the position of a node must be considered with other parameters during a forwarding node selection.

The typical CH nodes election and periodic changes of CHs in each round increase the total number of control messages to broadcast and to receive during the CH nodes election and clusters formation. It dissipates an additional energy of nodes throughout the network lifetime. The quasi-static period defined by the threshold energy level and table driven RC nodes election of EDR is more energy-efficient compared to conventional clustering-based data routing approaches. A table driven RC nodes election reduces broadcasting the number of control messages during the RC nodes election that saves the energy consumption of transmitting and receiving an additional number of control messages.

The network lifetime differs based on node distributions over the network. The protocols perform better in uniform node distribution compared to nonuniform, due to the balanced energy consumption of nodes. Although the uniform node distribution prolongs the steady state as well as the network lifetime, a part of distant nodes may isolate from the sink after a certain period of time, as in EDR and 3D EADC, because the nearest nodes may die more quickly than the distant nodes, as the nearest nodes are involved with additional data forwarding tasks compared to distant nodes. To address the problem, two solutions can be considered. Firstly, increase the number of node deployments near to the sink, so that the energy consumption of the nearest nodes would be more distributed. Secondly, an optimal *T*_E_ needs to be defined based on the local circumstances of the deployed nodes in different subspaces, as in the EDR.

The throughput depends on the steady state and network lifetime in these network topologies and routing policies. It increases with increases in the steady state and network lifetime. Therefore, an energy economical routing protocol design is a vital factor to prolong the steady state and network lifetime of an energy constraint 3D WSN.

## 5. Conclusions

In this paper, we propose the EDR protocol in a 3D WSN with uniform node distribution. An eccentricity region based RC nodes election algorithm is adopted that balances the energy consumption among member nodes by distributing equal numbers of nodes in the subspaces. The quasi-static feature of RC nodes election reduces a significant number of control messages during the election periods, so that a node can save the extra energy dissipation in this regard. The number of the messages is more reduced with a longer average quasi-static period of a node due to equal member nodes in the subspaces. A routing algorithm is also adopted for multi-hop communication. The imbalanced energy consumption of nodes caused by data forwarding tasks in particular is solved by selecting an RC node determined by the distance, residual energy and number of member nodes that balances the energy consumption among the forwarder RC nodes and reduces the number of hops towards the sink. With the benefit of the uniform node distribution, the EDR protocol is more robust to dominate such of the energy consumption factors of the constituted nodes that prolongs the steady state and network lifetime significantly.

We have performed extensive simulation to evaluate the performance of the protocol with uniform node distribution. The selected performance metrics for this analysis is mainly the steady state and network lifetime. Besides, the number of control packets, number of hops and throughput are considered. The simulation results show that the EDR protocol performs better for the selected metrics as compared to the existing protocols.

## Figures and Tables

**Table 1:** Description of control and data messages.

Message	Description
*Hello_Msg*	Tuple (*node_id, subspace_id, node_coordinates*), a control message used to collect the initial information of nodes during network initialization.
*Node_Msg*	Tuple (*node_id, subspace_id, average_distance, residual_energy, node_fitness*), a control message used to collect nodes’ information during RC nodes election.
*Schedule_Msg*	Tuple (*RC_id, subspace_id, [TDMA schedule]*), a control message used to assign the time slots to nodes during data communication.
*Route_Msg*	Tuple (*RC_id, subspace_id, node_coordinates, node_type, distance_sink, RC_fitness*), a control message used to collect RC nodes’ information during route selection towards the sink.
*Not_Msg*	Tuple (*RC_id, subspace_id, missing_id*), a notification message from an RC node to its member nodes when its residual energy gets as below as the threshold energy level. Meanwhile, it contains the *id* of missing member node(s).
*D_Msg*	Tuple (*node_id, subspace_id, RC_id, ‘local_data’*), a local data message from a member node to an RC node.
*AD_Msg*	Tuple (*RC_id, subspace_id, nexthop_id, [node_id, ‘fused_data’]*), an aggregated data message from an RC node to next hop/sink.

**Table 2:** Simulation parameters setting.

Parameter	Value
Sensor field	(200 × 200 × 200) m^3^
Sink position	(100, 100, 250) m
Number of sensor nodes: *n*	120
Subspace edge length: *SubSL*	100 m
Initial energy of a sensor node: *Ei*	0.5 J
Threshold energy: *T*_E_	*Ei*/*α*|(*α* = 6) = 0.08 J
Weight factor: *β*	0.5
Data packet size:	
*D_Msg*	500 bits
*AD_Msg*	4000 bits
Control packet size	256 bits
The transmitter or receiver circuitry: *E*_elec_	50 nJ/bit
Data aggregation cost: *E*_DA_	5 nJ/bit/report
Transmit amplifier cost: *E*_amp_ (*d* > *d*_0_)	0.0013 pJ/bit/m^2^
Transmit amplifier cost: *E*_fs_ (*d* ≤ *d*_0_)	10 pJ/bit/m^4^
Energy dissipation for sensing: *E*_sen_	0 J/bit
Threshold distance: *T*_D_	180 m
Maximum transmission range: *R*_max_	346.41 m
Transmission range equal to a subspace diagonal length: *R*_s_	173.20 m
Adjustable transmission range: *R*_min_	<*R*_s_
